# Nanomaterials for photothermal cancer therapy

**DOI:** 10.1039/d3ra02620e

**Published:** 2023-05-11

**Authors:** Shufan Duan, Yanling Hu, Ying Zhao, Kaiyuan Tang, Zhijing Zhang, Zilu Liu, Ying Wang, Haiyang Guo, Yuchen Miao, Hengda Du, Dongliang Yang, Shengke Li, Junjie Zhang

**Affiliations:** a Anhui Province Key Laboratory of Translational Cancer Research, School of Fundamental Sciences, Bengbu Medical College Bengbu 233030 China zhangjj@bbmc.edu.cn; b Nanjing Polytechnic Institute Nanjing 210048 China huyanling@njpi.edu.cn; c Department of Radiology, Nanjing First Hospital, Nanjing Medical University Nanjing 210006 China; d Key Laboratory of Flexible Electronics (KLOFE) and Institute of Advanced Materials (IAM), School of Physical and Mathematical Sciences, Nanjing Tech University (NanjingTech) Nanjing 211816 China yangdl1023@njtech.edu.cn; e State Key Laboratory of Quality Research in Chinese Medicine, Institute of Chinese Medical Sciences, University of Macau Taipa Macau SAR China

## Abstract

Cancer has emerged as a pressing global public health issue, and improving the effectiveness of cancer treatment remains one of the foremost challenges of modern medicine. The primary clinical methods of treating cancer, including surgery, chemotherapy and radiotherapy, inevitably result in some adverse effects on the body. However, the advent of photothermal therapy offers an alternative route for cancer treatment. Photothermal therapy relies on photothermal agents with photothermal conversion capability to eliminate tumors at high temperatures, which offers advantages of high precision and low toxicity. As nanomaterials increasingly play a pivotal role in tumor prevention and treatment, nanomaterial-based photothermal therapy has gained significant attention owing to its superior photothermal properties and tumor-killing abilities. In this review, we briefly summarize and introduce the applications of common organic photothermal conversion materials (*e.g.*, cyanine-based nanomaterials, porphyrin-based nanomaterials, polymer-based nanomaterials, *etc.*) and inorganic photothermal conversion materials (*e.g.*, noble metal nanomaterials, carbon-based nanomaterials, *etc.*) in tumor photothermal therapy in recent years. Finally, the problems of photothermal nanomaterials in antitumour therapy applications are discussed. It is believed that nanomaterial-based photothermal therapy will have good application prospects in tumor treatment in the future.

## Abstract 2

This review summarizes the common inorganic and organic photothermal nanoagents and their applications in tumor therapy. Additionally, the challenges and future prospects of nanomaterial-based photothermal therapy in cancer treatment are discussed.

## Graphical abstract

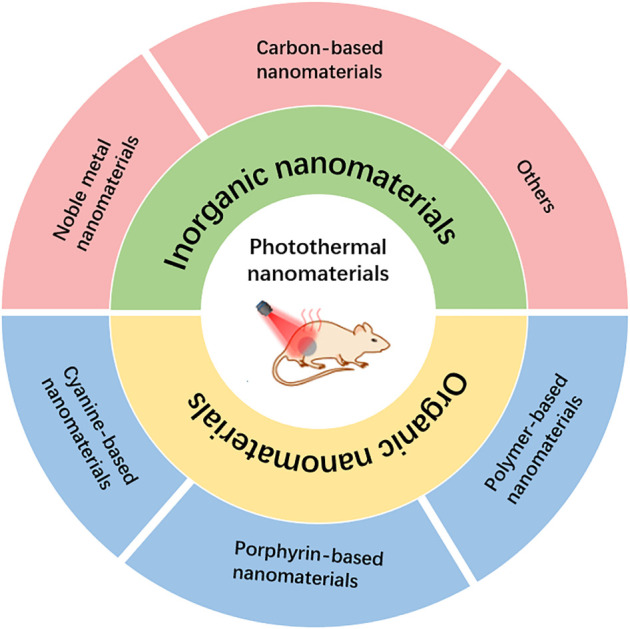

## 1. Introduction

Cancer remains a major contributor to global disease morbidity and mortality rates.^1^ According to statistics, an estimated 19.3 million cancer cases and 10 million cancer-related deaths were reported worldwide in 2020, and the incidence rates are still rising.^2^ Conventional cancer therapies, including surgery, radiation therapy and chemotherapy, inevitably cause collateral damage to healthy tissues, underscoring the need for novel approaches. As a result, nanomaterial-based photothermal therapy (PTT) has emerged as a promising solution, garnering considerable attention from researchers aiming to reduce the harm caused by cancer treatment, enhance the treatment efficacy, and improve therapeutic outcomes.

PTT is a promising therapeutic technique that utilizes photothermal materials, such as organic dyes or nanoparticles, to convert light energy into heat.^3,4^ This heat can selectively destroy cancer cells. The heat generated by these materials can be precisely controlled by adjusting the intensity and duration of light exposure, as well as the concentration and size of the photothermal materials.^5^ This selective treatment can minimize damage to healthy tissues, making it a minimally invasive technique that can be performed using non-invasive light sources such as lasers or light-emitting diodes (LEDs). PTT also has several advantages, including high selectivity and specificity, which allows for precise targeting of cancer cells.^6^ It can be used in combination with other therapeutic modalities, such as chemotherapy, radiation therapy (RT),^7^ chemodynamic therapy (CDT),^8^ photodynamic therapy (PDT),^9^ immunotherapy^10–12^ or gene therapy^13,14^ to enhance treatment outcomes. Despite these advantages, photothermal therapy has some limitations, including limited tissue penetration depth, low photothermal conversion efficiency and delivery efficiency, which can affect the therapeutic efficiency. Furthermore, the potential toxicity of photothermal materials used in photothermal therapy must be carefully considered and evaluated.

To achieve successful PTT, two requirements must be met: a near-infrared light (NIR) with superior tissue penetration and a photothermal nanoagent (PTA) that can induce photothermal effects.^15^ In the process of PTT, PTA is first administered through intravenous or alternative routes and selectively accumulates in the tumor tissue *via* the enhanced penetration and retention effect (EPR) characteristic of solid tumors. Subsequently, a high-penetration NIR laser is directed at tumor tissue, and PTA absorbs specific wavelengths of NIR energy and converts it into heat energy through nonradiative decay, rapidly elevating the local temperature of the tumor to a threshold that is lethal to tumor cells.^16,17^ Due to the PTA with specific photophysical properties, it can be used for tumor imaging, such as thermal imaging, fluorescence imaging (FL), and photoacoustic imaging (PAI), which has great potential for application in tumor visualization and treatment.^18,19^ The effectiveness of PTT largely depends on the photothermal conversion efficiency of PTAs.^16,20,21^ Therefore, it is critical to prepare PTAs with high photothermal conversion efficiency and strong NIR light absorption to achieve optimal PTT outcomes. Moreover, PTAs with good biocompatibility, tumor tissue-specific accumulation ability, and biodegradability are also essential properties for clinical application.

**Fig. 1:**
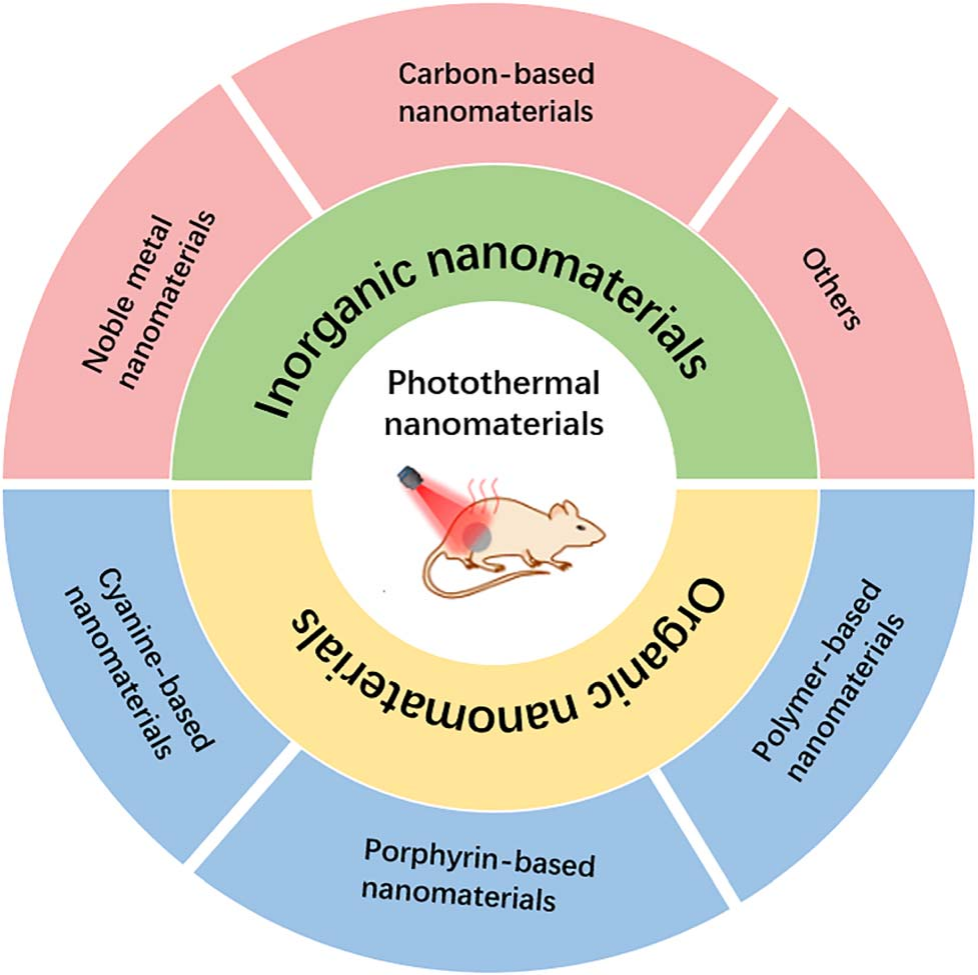
Schematic diagram of the classification of nanomaterial-based PTT.

In this review, we classify nanomaterials for PTT into two broad categories: organic nanomaterials and inorganic nanomaterials. Organic-based nanomaterials mainly include anatase-, porphyrin-, and polymer-based nanomaterials, and inorganic nanomaterials mainly include noble metal materials, graphene-based materials, copper-based sulfur nanomaterials, and other nanoagents (Fig. 1). Then, the challenges and prospects of the application of nanomaterial-based PTT in cancer are discussed. It is expected that with the continuous updating and development of nanotechnology, the application of nanomaterials in the biomedical field will also be more mature and perfect.

## 2. Organic nanomaterials

Organic nanomaterials offer superior photothermal properties, which make them promising applications in PTT and photothermal imaging for tumors. Progress has been made in designing organic PTAs with desirable photophysical and chemical properties. The development of organic nanomaterials has significantly improved the pharmacokinetics, targeting ability, photobleaching resistance and photothermal efficiency of conventional organic molecules. In this review, we highlight cyanine- and porphyrin-based nanomaterials, polymer-based nanomaterials, and other organic-based nanomaterials for the diagnosis and treatment of tumors.

### 2.1. Cyanine-based nanomaterials

**Fig. 2:**
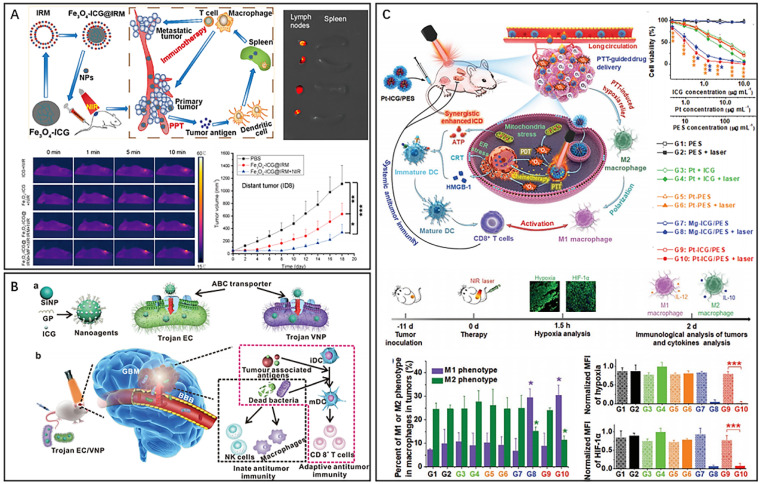
(A) Fe_3_O_4_-ICG@IRM for photothermal immunotherapy. Reproduced from ref. 28 with permission from ACS, copyright 2021. (B) Bacteria-based nanosystem for glioblastoma photothermal immunotherapy. Reproduced from ref. 31 with permission from Nature, copyright 2022. (C) Pt-ICG/PES for improving the antitumour immune response. Reproduced from ref. 35 with permission from Wiley, copyright 2022.

Cyanine dyes, which are composed of aromatic nitrogen-containing heterocycles linked by polymethyl chains, exhibit significant NIR absorption in the 700–900 nm range and are commonly used as PTAs, photosensitizers and fluorescent probes.^22–24^ Indocyanine green (ICG) is a FDA-approved, water-soluble dye that has been widely used as a contrast agent in medical imaging and as a photosensitizer in PDT.^25^ In recent years, ICG has also emerged as a promising organic photothermal conversion material for PTT.^26^ Therefore, ICG can be used for PTT/PDT combined therapy. However, free ICG is less stable and is generally not used alone in anticancer therapy.^27^ Xiong *et al.* developed bionanoparticles (Fe_3_O_4_-ICG@IRM) for synergistic thermoimmunotherapy by loading magnetic Fe_3_O_4_ nanoparticles with ICG and coating them with a hybrid membrane (IRM) composed of ID8 ovarian cancer cell membranes and red blood cell membranes (Fig. 2A).^28^ The bionic cytomembrane preserves the membrane proteins of ID8 cancer cells and red blood cells, improving the specific recognition of ID8 cells and blood circulation lifetime of the nanoparticles. Due to the excellent photothermal properties of ICG, the heat generated by PTAs can lead to tumor necrosis. The immunogenic cell death (ICD) induced by PTT was able to release tumor antigens, activate cytotoxic CD8^+^ T cells and reduce regulatory Foxp3^+^ T cells, effectively inhibiting the development of primary and metastatic tumors. However, due to the heterogeneous structure and abnormal vascular distribution in tumors, the low targeting and low penetration of nanomaterials greatly affects the uniformity of heat generated by ICG-mediated PTT, ultimately leading to tumor metastasis and recurrence.^6,29^ To address this limitation, a photothermal nanocluster (ICG@HSA-Azo-HP) was constructed by loading ICG onto human serum albumin (HSA), subsequently cross-linking it with a thermoresponsive azo linker (VA057), and modifying the specific homing/penetrating tLyP-1 peptide (HP).^30^ The nanoclusters (149 nm) were selectively aggregated at the tumor site by the EPR effect and then decomposed into small particles (11 nm) with enhanced intratumor diffusion ability under 808 nm laser irradiation. Meanwhile, HP activated the neuropilin-1 (NRP-1)-mediated endocytosis/extracellular transport pathway, further promoting the penetration of nanoclusters and ICG in tumors. In addition, a Trojan bacterium was designed for photothermal–immune combination therapy of glioblastoma (GBM) (Fig. 2B).^31^ The Trojan bacterium (GP-ICG-SiNPs) was composed of silica nanoparticles conjugated with glucose polymer (GP) and loaded with ICG. GP-ICG-SiNPs were internalized and stored by parthenogenic anaerobic bacteria *via* a specific ATP-binding cassette (ABC) transporter. Intravenous injection of this particular bacterium could easily carry nanomaterials to bypass the blood–brain barrier and target and penetrate GBM tissue. Under 808 nm laser irradiation, ICG molecules convert light energy into heat to destroy tumor cells and host bacteria, promoting the release of tumor-associated antigens (TAAs) and pathogen-associated molecular patterns (PAMPs), which facilitate the infiltration of CD8^+^ T cells and the activation of immune cells, such as macrophages and natural killer cells. Despite the good photodynamic properties of ICG, the application of ICG is often hindered by photobleaching and aggregation-caused quenching (ACQ).^32–34^ To overcome this issue, a novel nanoparticle (Pt-ICG/PES) was designed consisting of ICG, cisplatin, and the temperature-sensitive block polymer polypoly[2-(2-methoxyethoxy) ethyl methacrylate-co-poly (ethylene glycol) methyl ether methacrylate]-*b*-poly(sodium-*p*-styrenesulfonate) (PES) (Fig. 2C).^35^ PES acts as an isolator to control the aggregation level of ICG, allowing the complex to balance the ACQ effect and photobleaching for maximum photothermal conversion and singlet oxygen production of ICG. Throughout the therapeutic process, PTT dilates the blood vessels in tumor tissues and accelerates blood flow, thereby alleviating hypoxia in the tumor microenvironment and promoting the polarization of immunosuppressed M2 macrophages. Cisplatin-mediated chemotherapy and ICG-mediated PTT resulted in an increased release of danger-associated molecular patterns, leading to ICD and DC maturation. Ultimately, the combination therapy activated CD8^+^ T cells, resulting in a long-term systemic antitumour immune response.

Compared to other organic nanomaterials, such as polymer-based materials and porphyrins, ICG has several distinct advantages. Firstly, ICG has a high absorption coefficient in the near-infrared (NIR) region, which can enhance tissue penetration depth and minimizes light scattering in biological tissues. This allows for efficient conversion of light energy into heat and selective destruction of cancer cells. Secondly, ICG has low toxicity and high biocompatibility, which makes it suitable for using *in vivo*. In addition, ICG can be rapidly cleared from the body through the liver and kidneys. Thirdly, ICG has a relatively low cost and is commercially available, which facilitates its translation to clinical applications.

### 2.2. Porphyrin-based nanomaterials

Porphyrins are a group of macromolecular heterocyclic compounds formed by interconnecting the α-carbon atoms of four pyrrole-like substituents through hypomethyl bridges (=CH–).^36^ Porphyrins can be synthesized through various pathways, such as laboratory synthesis and *in vivo* synthesis. Natural porphyrins exist as the form of hemoglobin and hemocyanin in animals and play a crucial role in the aerobic respiration process.^37^ Porphyrin-based nanomaterials are a class of organic photothermal conversion materials that have attracted significant attention for their potential applications in PTT.^38^ One of the key features of porphyrin-based nanomaterials is their strong light absorption properties in the visible and near-infrared regions, which makes them suitable for using in PTT.^16,24,39^

**Fig. 3:**
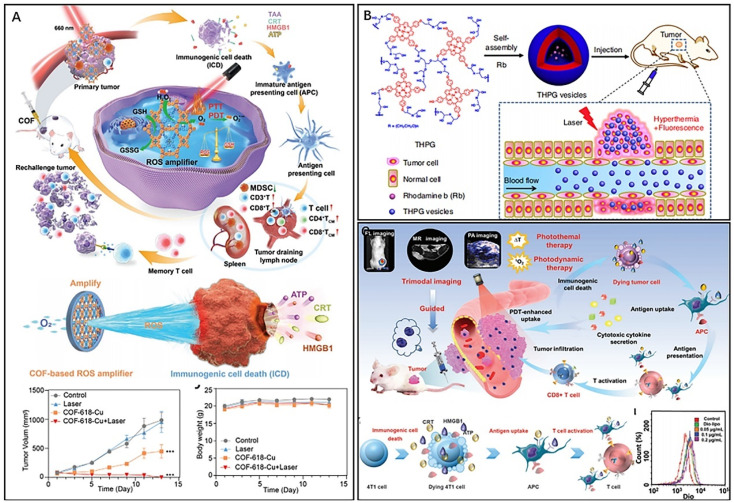
(A) COF-618-Cu for remodelling TME and immunotherapy. Reproduced from ref. 41 with permission from Wiley, copyright 2022. (B) Polymer vesicles for imaging-guided PTT. Reproduced from ref. 45 with permission from Nature, copyright 2020. (C) Versatile nanoporphyrin Pp18-lipos for multimodal imaging and combined therapy. Reproduced from ref. 46 with permission from Wiley, copyright 2020.

Zhou *et al.* developed two-dimensional gold–porphyrin-based nanosheets, Au0–Por, with outstanding photothermal properties and high drug loading capability by mixing tetrachloroaurate and meso-tetra(pyridyl)porphyrin (TPyP) solutions and reducing gold ions.^40^ Nanosheets loaded with carbon monoxide-releasing molecules (CORMs) respond to overexpressed hydrogen peroxide in tumors and produce carbon monoxide for gas therapy. Additionally, gold atoms exhibited excellent glucose oxidase-like activity, consuming glucose and increasing hydrogen peroxide, which further enhanced the release of carbon monoxide in a cascade. *In vivo* experiments have shown that Au0–Por nanosheets effectively inhibit tumor growth in combination therapy guided by PAI and FL. Moreover, the nanosheets could be degraded and excreted through the kidneys. To achieve the desired phototherapeutic effect, porphyrin-based nanomaterials must overcome photobleaching and ACQ effects and counteract the malignant microenvironment of tumors, such as hypoxia and overexpression of glutathione (GSH). Zhang *et al.* developed a novel staggered stacked covalent organic framework (COF-618-Cu) for synergistic phototherapy and immunotherapy by covalently bonding porphyrin monomers to the backbone of the covalent organic framework (Fig. 3A).^41^ The staggered stacked and porous structure of COF-618-Cu maintained the spatial density of porphyrin monomers, effectively reducing the ACQ effect. Furthermore, the catalase-like and peroxidase-like activities of COF-618-Cu generated oxygen and amplified the killing effect of reactive oxygen species (ROS). *In vivo* studies have demonstrated that COF-618-Cu-mediated synergistic PDT and PTT induced ICD and the release of damage-associated molecular patterns (DAMPs), such as adenosine triphosphate (ATP), calreticulin (CRT), and high mobility histone B1 (HMGB1). ICD also promotes the proliferation and differentiation of effector and memory T cells and reduces immunosuppressive cells, facilitating the remodelling of the tumour microenvironment and the maintenance of long-term antitumour immune responses. In addition, supramolecular structures based on porphyrins have been developed to enhance the optical properties and promote penetration and killing in deep tumors.^42–44^ A nanoscale polymer vesicle composed of hyperbranched polyporphyrins has been reported (Fig. 3B).^45^ The porphyrin monomer further self-assembled into nanofilamentous supramolecular polymers driven by π–π stacking, resulting in superior photothermal conversion (44.1%). Moreover, the nanovesicles could encapsulate functional molecules such as rhodamine b (RbB) and Cy7.5 for fluorescence imaging. Sun *et al.* obtained multifunctional liposome-like nanoporphyrins (Pp18-lipos) by the self-assembly of lipid–purpurin 18 conjugates and pure lipids (Pp18-lipids) (Fig. 3C).^46^ Pp18 is a chlorophyll derivative that has been used as a photosensitizer for the treatment of malignant tumors. By adjusting the dosage of Pp18, Pp18-lipos with different physicochemical properties can be obtained; for example, nanoporphyrin containing 2 mol% Pp18 could be used for PDT and FL, and nanoporphyrin containing 65 mol% Pp18 could be used for PTT and PAI. Furthermore, nanoporphyrin has excellent T1-weighted magnetic resonance imaging (MRI) by chelating Mn^2+^. *In vivo* experimental results demonstrate that Pp18-lipos with two types of compositions have excellent synergistic efficacy in PTT and PDT. The combination treatment achieved 90.1% tumor inhibition in subcutaneous 4T1 tumor-bearing mice at a dose of 6 mg kg^−1^ Pp18.

Porphyrin-based nanomaterials can be easily functionalized with targeting ligands or therapeutic agents, which is important for clinical applications. In addition, porphyrin-based nanomaterials have good biocompatibility and biodegradability, making them suitable for using in the human body. They also have a high photothermal conversion efficiency, which allows for effective destruction of cancer cells at relatively low concentrations. However, porphyrin-based nanomaterials also have some limitations. For example, they may have limited tissue penetration depth due to their absorption properties in the visible and near-infrared I regions. Additionally, the stability and biocompatibility of porphyrin-based nanomaterials can be affected by environment factors such as pH, temperature, and enzymatic degradation.^47^ Overall, porphyrin-based nanomaterials are a promising class of organic photothermal conversion materials. However, further research is needed to optimize their properties and overcome their limitations for broader clinical applications.

### 2.3. Polymer-based nanomaterials

**Fig. 4:**
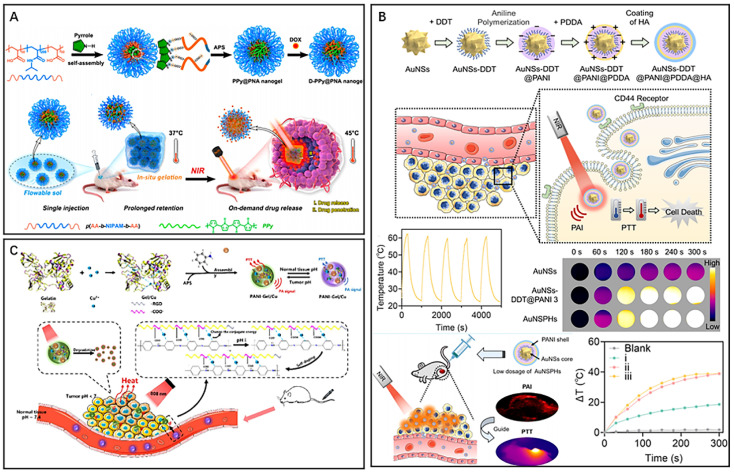
(A) D-PPy@PNA nanogel for synergistic phototherapy and chemotherapy. Reproduced from ref. 51 with permission from ACS, copyright 2020. (B) AuNSPHs for laser-induced tumor ablation. Reproduced from ref. 61 with permission from ACS, copyright 2022. (C) PANI–Gel/Cu for pH-responsive PTT. Reproduced from ref. 62 with permission from ACS, copyright 2022.

Polypyrrole (PPy), polydopamine (PDA) and polyaniline (PANI) are commonly used organic photothermal materials with a wide range of applications.^48^ PPy exhibits high stability, biocompatibility and excellent photothermal conversion ability,^49^ and polypyrrole also has good application in photoacoustic imaging of tumors.^50^ Geng *et al.* synthesized temperature-sensitive nanogels D-PPy@PNA by intersorbing pyrrole monomers with poly (acrylic acid-*b-N*-isopropylamide-*b*-acrylic acid) (PNA) using the principle of acid-base neutralization, followed by adding the oxidant ammonium persulfate (APS) to polymerize pyrrole monomers and finally load DOX (Fig. 4A).^51^ The introduction of PNA improved the colloidal stability of PPy and achieved temperature-responsive release of DOX *in vivo* due to its excellent temperature sensitivity. After injection of the nanosol into mice, a hydrogel formed and remained in a long-term retention state. Upon NIR irradiation, the PPy component of the nanogel generated a large amount of heat for PTT with the thermogenic phase change of the hydrogel at the lesion. The drug DOX was released after the phase change of the hydrogel, which was enhanced by NIR irradiation and exhibited excellent penetration at the tumor. Experiments demonstrated that D-PPy@PNA has biocompatibility and exhibits PTT-sensitizing chemotherapy in the treatment of tumors, which synergistically enhances the therapeutic efficacy. PDA is a versatile organic nanophotothermal conversion material that is widely used as a multifunctional nanotherapeutic platform.^52^ The PDA with excellent photothermal conversion ability, ease of surface modification and other properties such as antibacterial activity, free radical scavenging, and UV shielding, makes it a popular choice for various biomedical applications.^53–55^ Li *et al.* developed magnetic nanocomplexes (Fe_3_O_4_@PDA) for MRI-guided cancer PTT by wrapping Fe_3_O_4_ nanoparticles with PDA.^56^ The magnetic Fe_3_O_4_ nanoparticles endowed the nanocomplexes with MRI capability and enabled magnetothermal therapy of tumors. The PDA had ideal photothermal properties and was applied to tumor PTT while improving the efficacy of magnetothermal therapy. Moreover, the PDA coating imparts excellent stability and biocompatibility to the nanomaterials, making them long-term MRI contrast agents. Shao *et al.* successfully constructed core–shell nanoparticles composed of PDA and hollow mesoporous silica and used them for effective combination therapy (PDA@hm@CQ@GO_*x*_) by loading chloroquine (CQ) and modifying glucose oxidase (GO_*x*_) to inhibit tumor proliferation.^57^ CQ inhibits tumor autophagy and disrupts tumor cell self-homeostasis, while GO_*x*_ mediated starvation therapy can inhibit tumor cell growth and the expression of heat shock proteins (HSPs), further effectively reducing the ability of tumors to repair thermal damage. *In vivo* experiments have shown that the nanocomplexes compensate for the anticancer effect of mild PTT by inhibiting autophagy and regulating glucose metabolism, thereby achieving an enhanced combined therapeutic effect. PANI is another commonly used organic photothermal material due to its excellent stability, biocompatibility, and excellent NIR absorption, making it ideal for PTT and PAI.^58,59^ In addition, due to the low cytotoxicity of PANI, the combination of PANI and metallic nanomaterials could improve conversion efficiency while reducing metal toxicity due to the low cytotoxicity of PANI.^60^ Wang *et al.* developed nanoprobes (AuNSPHs) for PAI-guided PTT by using 1-dodecylmercaptan (DDT)-modified gold nanostars (AuNSs) and modified PANI, poly (diallyldimethylammonium chloride) (PDDA) and hyaluronic acid (HA) on their surfaces in a stepwise manner (Fig. 4B).^61^ The combination of PANI and AuNSs resulted in higher photothermal conversion ability, and the optimal PANI shell thickness for AuNSPHs was found to be 20 nm. The results from experiments showed that a single injection of low-dose AuNSPHs could effectively inhibit tumor growth. These nanoprobes, with excellent biocompatibility, functioned as a safe and efficient targeted PTA, which showed an enhanced therapeutic effect of laser-induced tumor ablation in PAI-guided PTT. Li *et al.* obtained nanocomplexes (PANI–Gel/Cu) for targeted PTT of tumors by mixing aniline solution with gelatin-Cu^2+^ complexes under the induction of ammonium persulfate (Fig. 4C).^62^ The high hydrophilicity of gelatine and the RGD sequence increased the enrichment of the nanocomplexes in tumour tissues. Moreover, the acidic tumor microenvironment promoted the self-doping effect of the carboxyl group in gelatin with the imine part of polyaniline, which led to improved photothermal conversion efficiency of the nanomaterials and activated PAI. The results from *in vivo* experiments showed that the acidity-triggered photoacoustic signal of PANI–Gel/Cu could be used for the visualization of photothermal ablation of tumors, thereby demonstrating a promising pH-responsive nanotherapeutic platform. Polymer-based nanomaterials can be designed with tunable photothermal conversion properties by adjusting the composition, size, and shape of the polymer and incorporating different chromophores or metals. However, polymer-based nanomaterials also have some limitations. One of the main challenges is to achieve high photothermal conversion efficiency while maintaining good biocompatibility and biodegradability. The polymer structures and surface chemistry can affect the stability and photothermal conversion efficiency of polymer-based nanomaterials, and their properties may be sensitive to environmental factors such as temperature, pH, and ionic strength.

### 2.4. Other organic nanomaterials

**Fig. 5:**
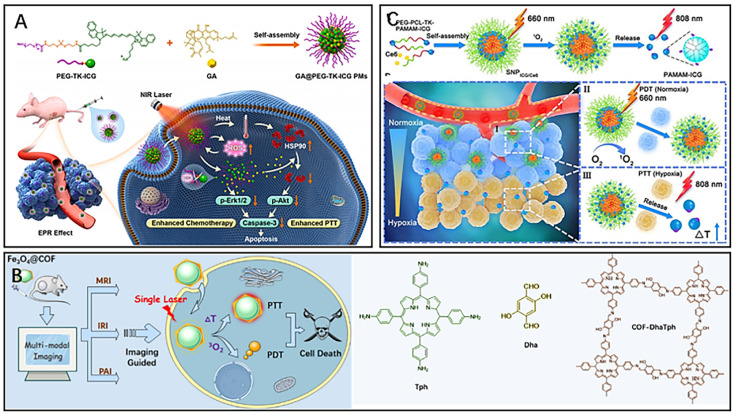
(A) The principle of GA@PEG-TK-ICG PMs for photothermal/chemotherapy combined therapy. Reproduced from ref. 71 with permission from Elsevier, copyright 2021. (B) Fe_3_O_4_@COFDhaTph for phototherapy and multiple-model imaging. Reproduced from ref. 77 with permission from ACS, copyright 2021. (C) The principle of tumor localization phototherapy of SNP_ICG/Ce6_. under near-infrared irradiation. Reproduced from ref. 79 with permission from ACS, copyright 2020.

In biological applications, organic nanocarriers are favoured for their low toxicity, ideal drug-carrying capacity, high biocompatibility, and *in vivo* degradability, which in combination with PTT can effectively inhibit malignant tumour proliferation and metastasis.^63–65^ Polymeric micelles (PMs), composed of amphiphilic copolymers with different blocks, are spherical colloidal particles capable of loading poorly water-soluble drugs. PMs allow for responsive drug release by adjusting the hydrophilic or hydrophobic blocks of the copolymer, thereby improving the drug utilization rate.^66,67^ Yang *et al.* formed an amphiphilic polymeric micelle (PEG-TK-ICG PMs) by linking hydrophobic ICG and hydrophilic polyethylene glycol (PEG) through ROS-responsive thioketal (TK) bonds^68–70^ and then loaded the chemotherapeutic drug garcinia cambogia (GA) into the polymeric micelle to finally prepare a nanosystem (GA@PEG-TK-ICG PMs) with combined photothermal/chemo combined therapeutic effects (Fig. 5A).^71^ In this nanosystem, the fluorescent dye ICG could not only act as a PTA to exert a PTT effect under NIR irradiation but also act as a photosensitizer to absorb light energy to generate a large amount of ROS for breaking TK bonds to disintegrate polymeric micelles, thus releasing GA selectively in tumor tissues. While the release of the natural compound GA could inhibit the expression of phosphorylated extracellular regulated protein kinases (p-Erk1/2) and thus exert chemotherapeutic effects to inhibit tumor growth, GA could also downregulate the overexpression of heat shock protein (HPS90) and subsequently inhibit phosphorylated protein kinase B (p-Akt), thereby improving the efficiency of photothermal therapy. Thus, these polymeric micelles (GA@PEG-TK-ICG PMs) loaded with GA exerted specific photothermal/chemo combined therapy under near-infrared light irradiation. Liposomes are self-assembled vesicles composed of phospholipid bilayers, offering high biocompatibility, nontoxicity, and nonimmunogenicity.^72^ These structures can encapsulate both hydrophilic and hydrophobic compounds and have found applications in the diagnosis and treatment of tumors. Chen *et al.* constructed a liposome-hydrogel system (IR780/Lipo/gel) for topical skin delivery using liposomes (Lipo), the photosensitizer IR780, and a hydrogel (gel).^73^ IR780 is a lipophilic organic dye that can target tumor cells without the need for additional chemical modification.^74^ Hydrogels, which are highly hydrophilic, are incorporated into liposomes to achieve local targeting of superficial tumors. In *in vivo* experiments, the liposome-hydrogel system was able to deliver IR780 into the tumor tissues through the skin barrier *via* local administration, further resulting in enhanced therapeutic effects of PTT.

Covalent organic frameworks (COFs) are emerging as promising organic crystalline nanomaterials with unique porous properties, tunable porosity, and high biocompatibility.^75,76^ COFs have been widely used for combined tumor therapy and multimodal imaging. Feng *et al.* designed a novel nanocomposite (Fe_3_O_4_@COF-DhaTph) with a core–shell structure based on the unique properties of COF for tumor PTT/PDT combined therapy under single laser irradiation (Fig. 5B).^77^ The magnetic and optical properties of the nanocomposite make it possible to enhance the contrast of MRI, thermal imaging and PAI, thereby achieving multimodal imaging of tumors. The COF contains porphyrins, which can generate ROS for tumor PDT under laser irradiation. Magnetic iron oxide (Fe_3_O_4_) wrapped inside the COF significantly enhanced the photothermal conversion efficiency of the composite, which exhibited good photothermal ablation ability. Notably, the wavelengths of NIR light absorbed by the nanocomposite for the excitation of PDT and PTT have overlapping regions. Thus, using a single wavelength of NIR could activate PDT/PTT simultaneously, further significantly improving the efficiency of phototherapy. Dendrimers are highly controllable and versatile nanomaterials with a large number of multivalent groups on their surface, making them promising for drug and gene delivery.^78^ Wang *et al.* conjugated poly(amidoamine) dendrimer-conjugated ICG(PAMAM-ICG) with the amphiphilic polymer poly(ethylene glycol)-*b*-poly(ε-caprolactone) (PEG-*b*-PCL) by a singlet oxygen-responsive (TK) bond, and then the photosensitizer chlorin e6 (Ce6) was loaded into the polymeric nanomaterials to finally prepare a near-infrared light responsive material with size tunability and strong tumor penetration capability for targeted tumor phototherapy (SNPICG/Ce6) (Fig. 5C).^79^ The advantage of this polymeric material over conventional photothermal nanomaterials was that it overcame the inhibitory effect of the hypoxic tumor microenvironment on the antitumour effect of phototherapy. Because of the large size of SNPICG/Ce6, it could only accumulate in relatively oxygen-rich tumor tissues around blood vessels through the EPR effect after intravenous injection. Under 660 nm NIR irradiation, the photosensitizer Ce6 absorbed light energy to generate ROS for PDT, and the high concentration of ROS could break the TK bond, thus disintegrating the polymer SNPICG/Ce6 and releasing the small molecule PAMAM-ICG. PAMAM-ICG was able to penetrate into deep hypoxic tumor tissues due to its small size and exerted PTT efficacy under 808 nm NIR irradiation. Therefore, SNPICG/Ce6 was able to achieve deep PDT of tumors *via* an adjustable diameter strategy.

## 3. Inorganic nanomaterials

### 3.1. Noble metal nanomaterials

The utilization of precious metal nanomaterials such as gold, silver, platinum (Pt), and palladium has gained tremendous interest in the field of PTT due to their high absorption in the NIR region, strong photothermal conversion efficiency, robust local surface plasmon resonance (LSPR) effects, and synthetic tunability.^5,24,80^ These unique properties make precious metal materials highly suitable for inorganic PTAs. In addition, similar to organic PTAs, these inorganic PTAs can also be combined with other materials or drugs to form a novel nanocomplex. The multimodal nature of these nanocomplexes makes them highly effective in the treatment of tumors by combining different therapeutic modalities, thus providing an improved therapeutic outcome.

**Fig. 6:**
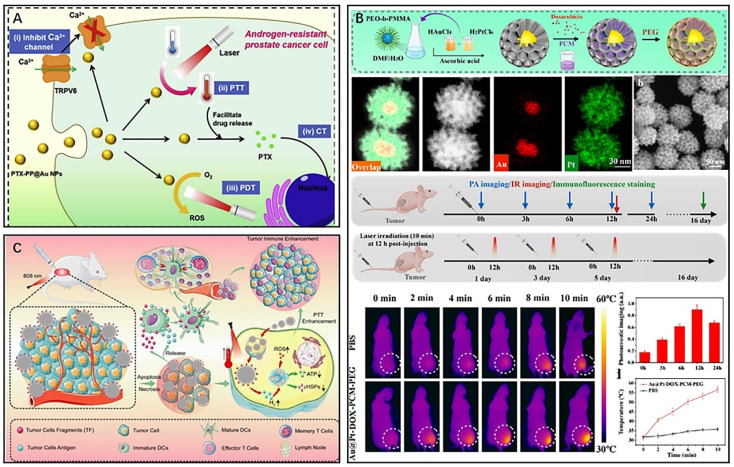
(A) Schematic diagram of the principle of antitumour therapy of PTX-PP@Au NPs. Reproduced from ref. 84 with permission from Elsevier, copyright 2019. (B) Au@Pt-DOX-PCM-PEG for hypoxia relief and combined therapy. Reproduced from ref. 89 with permission from ACS, copyright 2022. (C) A multifunctional PdHs-TF nanosystem for immunotherapy and PTT. Reproduced from ref. 93 with permission from Wiley, copyright 2022.

Among various precious metal nanomaterials, gold-based nanomaterials are the most extensively studied PTAs. Upon irradiation with light of a specific wavelength, the light absorption of gold nanoparticles resonates with the wavelength of the incident light, resulting in polarization of free conduction band electrons on the surface, *i.e.*, the occurrence of LSPR.^24^ At this point, gold nanoparticles exhibit enhanced light absorption and charge separation properties, which can effectively promote the conversion of light energy to thermal energy.^81^ To combine PTT and other therapeutic modalities, gold nanomaterials can also be integrated with other materials or drugs to form a novel nanocomplex that is well suited for multimodal treatment of tumors. Zhang *et al.* prepared a novel nanocarrier (AuHNRs-DTPP) by coupling a chimeric peptide (DTPP) containing the photosensitizer PpIX with Au hollow nanorods (AuHNRs), which exhibited a desirable PTT effect in the NIR-II window.^82^ Compared to laser irradiation in the NIR-I window, light in the NIR-II window has a higher tissue penetration ability and can perform more complete tumor photothermal ablation.^83^ Under 1064 nm laser irradiation, the nanocarrier could rapidly generate heat to achieve efficient PTT and then release the activated photosensitizer PpIX, which leaves the surface of the nanocarrier with enhanced fluorescence for real-time *in vivo* apoptosis imaging and could assist in PDT to kill cancer cells that survive PTT. The results of *in vivo* experiments showed significantly better antitumour effects in mice injected with AuHNRs-DTPP and irradiated with dual lasers (1060 and 633 nm). After serum examination, all the detected indexes were within the normal range, indicating that AuHNRs-DTPP had no significant hematotoxicity during treatment. However, during the preparation of gold nanocarriers, the toxicity of exogenous reducing agents was significant, and the gold carriers were unstable and had low drug loading efficiency. Additionally, the different radiation conditions of PTT or PDT also caused significant damage to the skin. Wang *et al.* reported on a novel polymeric delivery nanoparticle system (PTX-PP@Au NPs) loaded with paclitaxel (PTX) and gold cages for the treatment of lethal androgen-resistant prostate cancer (ARPC) *via* synergistic PTT/PDT/chemotherapy and TRPV6 channel inhibition (Fig. 6A).^84^ The researchers assembled Pluronic-polyethyleneimine into micelles and then wrapped the chemotherapeutic drug PTX to form PTX-PP NPs, which effectively controlled drug release and blocked the tumor cell cycle. The Au cage on the surface of PTX-PP NPs was designed to modify androgen resistance by blocking the TRPV6 cation channel, which inhibited tumor cells in ARPC. Furthermore, the Au cage provides stability to the nanosystem and acts as a photosensitizer for PTT/PDT. Due to the wide absorbance range, the nanosystem could generate heat and induce ROS production when irradiated with a single 808 nm NIR, making it useful for PTT combined with PDT for tumors, avoiding radiation damage to the skin caused by multiple NIR irradiations. Additionally, the nanosystem with good tumor targeting ability could promote the release of the chemotherapeutic drug PTX in tumor tissues as the temperature increased, further providing better therapeutic effects.

Gold-based nanomaterials have been widely used in tumor PTT.^85–87^ However, due to their low melting point, gold-based nanomaterials have the tendency to melt into other shapes under NIR irradiation, which deviates from the established target absorption region and reduces their photothermal conversion efficiency.^24^ In comparison, Pt not only possesses excellent photothermal conversion ability and a higher melting point but also functions as a catalyst in the tumor microenvironment.^88^ To combine the unique properties of both precious metals, Sun *et al.* synthesized a porous Au@Pt gold-platinum bimetallic nanocore-shell structure with excellent photothermal conversion properties and catalytic performance (Fig. 6B).^89^ They further developed a nanotherapeutic agent, Au@Pt-DOX-PCM-PEG, based on the Au@Pt bimetallic structure, which combined PTT with chemotherapy and reduced hypoxia-induced chemoresistance. The chemotherapeutic drug DOX was loaded into the Pt porosity by using the temperature-sensitive material lauric acid (PCM) as the seal material. The presence of the temperature-sensitive material lauric acid (PCM) improved the targeted release ability of DOX, while Pt, as an enzymatic catalyst, is able to catalyze the breakdown of endogenous hydrogen peroxide in tumors to produce oxygen to improve tumor microenvironment hypoxia. Pt also reduces the external transport of the chemotherapeutic drug DOX by inhibiting the expression of hypoxia-inducible factor-1a (HIF-1a), multidrug resistance gene 1 (MDR1), and P-gp to promote the accumulation of the chemotherapeutic drug in tumor cells. The Pt shell surface wrapping with polyethylene glycol (PEG) prolongs the circulation time of this nanotherapeutic agent in the blood. Additionally, the Au core absorbs NIR-II (1064 nm) laser light and triggers the release of DOX. In conclusion, this new nanocomplex was a combination of phototherapy and chemotherapy as an oxygen generator that significantly reduced the development of multidrug resistance in tumors. The use of platinum bimetal not only increases the melting point of the material but also has catalytic properties for improving tumor microenvironment hypoxia.

However, conventional PTT often induces the expression of heat shock proteins (HSPs), which decreases tumor sensitivity to hyperthermia and reduces the effectiveness of PTT treatment.^90–92^ To overcome this issue, Chen *et al.* designed a novel nanomaterial (PdHs-TF) by using metallic palladium as a raw material for tumor immunotherapy, gas therapy, and PTT (Fig. 6C).^93^ The PdHs efficiently loaded tumor fragment tumor antigen (TF) as an antigen, and under 808 nm laser irradiation, the TF was released and bound to dendritic cells, further activating the immune response *in vivo*. Moreover, the hydrogen atoms loaded in PdHs-TF could be released as hydrogen gas under laser irradiation to inhibit the expression of HSPs in tumor tissues, reducing the resistance of tumor tissues to high temperature. The released hydrogen gas diffuses freely between cells, disrupting the redox homeostasis of tumor cells and inducing oxidative stress^94–97^ and intracellular ROS production, thereby impeding mitochondrial respiration^98^ and reducing adenosine triphosphate (ATP) production, inhibiting tumor metastasis and spread, and improving the therapeutic effect of PTT.^99^ Additionally, PTT mediated by Pd in this nanomaterial could induce ICD of tumor cells, which further enhanced immune cell infiltration in TEM and improved the efficacy of tumor immunotherapy. Local thermal therapy can also reverse the immunosuppressive effects of TEM, enhancing immunity.^100,101^ Although precious metal nanomaterials have shown great potential, there are still issues and defects that need to be improved. Thus, researchers continue to develop more multifunctional nanocomposites based on precious metal materials to enhance their application potential in the treatment of tumors.

### 3.2. Carbon-based nanomaterials

Carbon-based nanomaterials, including graphene-based nanomaterials (GBNMs) and carbon nanotubes (CNTs), are commonly used as photothermal agents in inorganic nanomaterials.^102–104^ They also have potential applications in tumor treatment and antimicrobial therapy. However, their therapeutic effect as PTAs for tumors is often limited due to their inherent properties.^105^ To overcome this, researchers have used different functionalized groups to modify carbon-based nanomaterials to expand their application in PTT.

**Fig. 7:**
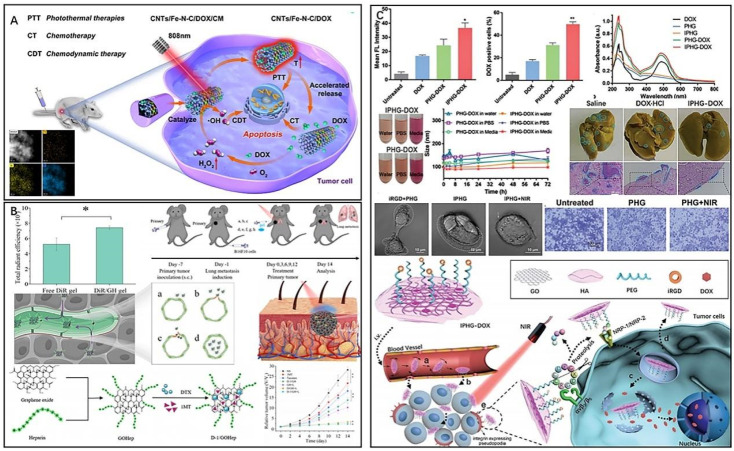
(A) CNTs/Fe–N–C/DOX/CM for the targeted treatment of breast cancer. Reproduced from ref. 107 with permission from ACS, copyright 2022. (B) D-1/GH for promoting drug infiltration and combined therapy. Reproduced from ref. 115 with permission from Elsevier, copyright 2022. (C) Multifunctional nanocomposite IPHG for the treatment of metastatic tumors. Reproduced from ref. 117 with permission from Wiley, copyright 2021.

CNTs are known for their stability, flexibility, and bioavailability, making them attractive for PTT applications. By modifying CNTs, researchers have been able to combine PTT with other therapies to improve cancer treatment.^106^ Han *et al.* synthesized CNTs/Fe–N–C by loading single-site Fe–N–C onto CNTs and prepared nanocomposites CNTs/Fe–N–C/DOX/CM for targeted treatment of breast cancer by encapsulating mesoporous CNTs/Fe–N–C with breast cancer cell membranes and loading DOX (Fig. 7A).^107^ Encapsulating cancer cell membranes enhances the tumor targeting ability of the drug and reduces drug efflux.^79,108,109^ Furthermore, CNTs/Fe–N–C with peroxidase (POD)-like activity could catalyze the generation of cytotoxic hydroxyl radicals (·OH) from H_2_O_2_ in the tumor microenvironment for chemodynamic therapy (CDT) of tumors. Additionally, the nanocomposites exhibited a photothermal effect that accelerated the release of DOX, and the released DOX could induce the production of endogenous H_2_O_2_ in tumors, achieving the combined therapeutic effect of PTT, CDT and chemotherapy. Zhao *et al.* prepared a nanovector CNT-PS/siRNA for combined PTT and gene therapy by coating sucrose laurate (SL) and peptide lipids (PL) on CNTs and loading anti-survivin siRNA.^110^ Due to the excellent photothermal conversion efficiency of CNTs, tumor cells can be killed by high temperature under NIR light irradiation. Meanwhile, SL and PL, with excellent temperature sensitivity, decomposed and released anti-survivalin siRNA at high temperature, which could inhibit the expression of tumor cell survival, thus exerting the effect of gene therapy. In addition, the prepared nanomaterials have good biocompatibility, affirming the prospect of combining PTT and gene therapy for antitumour therapy. However, the use of CNTs poses a potential risk to human health, as exposure to these materials has been reported to trigger local or systemic inflammatory responses. In addition, prolonged exposure to CNTs in the lungs may promote breast cancer metastasis.^111,112^

Graphene-based nanomaterials, with excellent physicochemical properties, have shown promise in the PTT of tumors. Surface modification can significantly reduce their immunotoxicity.^113,114^ Du *et al.* developed a novel approach for the treatment of melanoma using graphene oxide (D-1/GH) loaded with docetaxel (DTX) and 1-methyl-d-tryptophan and modified with heparin (Hep) (Fig. 7B).^115^ Hep has a high affinity for tumor tissue and can mediate the hydration of the stratum corneum. As a result, D-1/GH modified with Hep exhibited a good transdermal effect and enhanced tumor infiltration ability, improving drug penetration into the skin and promoting drug tumor accumulation in tumors. The introduction of the chemotherapeutic drug DTX provided D-1/GH with the therapeutic effect of PTT and chemotherapy. Additionally, 1MT inhibits CD8^+^ T-cell depletion and upregulation of Tregs by inhibiting indoleamine 2,3-dioxygenase (IDO) activity in tumors, thereby blocking tumor immune evasion. Therefore, D-1/GH achieved multiple therapeutic effects of PTT, chemotherapy, and immunotherapy while exhibiting enhanced tumor infiltration ability. Targeted delivery of antitumour agents can similarly enhance tumor infiltration and effectively improve drug efficacy while reducing drug toxicity.^116^ Wang *et al.* developed a graphene oxide (GO) nanosheet (IPHG) modified by HA, PEG and iRGD for the treatment of metastatic tumors (Fig. 7C).^117^ The nonspherical nanocomposites exhibited flip-flop motion in the bloodstream, and the disk-shaped shape gave IPHG a large aspect ratio. Surface modification of PEG and HA avoided unnecessary physiological aggregation of IPHG in the bloodstream, making the doxorubicin (DOX)-loaded drug formulation (IPHG-DOX) more accessible to the vascular endothelium. iRGD, a cyclic peptide with tumor-targeting and tumor-penetrating abilities, specifically recognizes and binds to integrins located on the surface of tumor cells, thereby assisting DOX in effectively infiltrating tumor cells and blocking signals for cell migration, thereby stifling primary tumor metastasis. *In vivo* experiments showed that IPHG-DOX-infiltrated tumor cells induced PTT and thermotherapeutic stress upon NIR stimulation, accelerated DOX release, and exhibited sensitization to chemotherapy and enhanced tumor suppression. Additionally, the high temperature caused by NIR irradiation inhibited tumor cell migration. IPHG not only serves as a carrier for drug shipment but also plays an important role in enhancing drug infiltration at tumor sites and PTT combined with integrins for the treatment of metastatic tumors. Huang *et al.* synthesized a novel pH-responsive multifunctional nanocomposite (GO-Fe_3_O_4_@Au@Ag-MPBA-DOX) for surface-enhanced Raman spectroscopy (SERS) by depositing Fe_3_O_4_@Au@Ag nanoparticles on graphene oxide (GO) and labelling the Raman reporter molecule 4-mercaptophenylboronic acid (4-MPBA).^118^ Doxorubicin (DOX) was attached to the PBA end of 4-MPBA through a borate ester bond, resulting in high DOX loading efficiency (25.6%) and encapsulation efficiency (82.7%) due to the π–π stacking and hydrophobic interactions between GO and DOX. The GO stabilized the surface-enhanced Raman spectroscopy (SERS) signal, while Fe_3_O_4_@Au@Ag nanoparticles enabled MRI. In an acidic tumor microenvironment, the pH-sensitive borate ester bond broke, releasing DOX, which was monitored in real time by changes in the 4-MPBA SERS spectra. Moreover, the free PBA actively bound to sialic acid residues on tumor cells, ensuring targeted delivery. Compared to high laser power PTT, mild PTT can effectively reduce the side effects induced by heat diffusion. However, the treatment efficiency of mild PTT is often reduced due to the cell self-repair ability. On the other hand, GO nanoparticles possess excellent biosafety properties and have been shown to enhance the efficacy of mild PTT when used in combination with HSP inhibitors. Chang *et al.* developed a multimodal imaging-guided mild photothermal therapy (PTT) agent by synthesizing a diagnostic agent (GO/BaHoF5/PEG/NVP-AUY922) after BaHoF5 nanoparticle-functionalized GO was modified with PEG and loaded with NVP-AUY922.^119^ The loaded NVP-AUY922 acted as an HSP90 inhibitor to enhance the sensitivity of tumor cells to heat therapy. The prepared GO/BaHoF5/PEG nanocomposites exhibited enhanced permeability and retention effects, easily accumulating at tumor sites. BaHoF5 nanoparticles endowed the agent with a high magnetic field MR/CT dual-modality bioimaging function, providing more accurate image information for tumor diagnosis and treatment. Compared to high laser power PTT, mild PTT combined with HSP inhibitors can reduce heat diffusion-induced side effects, and the efficacy can be improved using graphene oxide nanoparticles.

### 3.3. Other inorganic nanomaterials

In addition to precious metals and carbon-based nanomaterials, transition metal materials are a common class of PTAs that can be used in PTT for tumors.^120^ Among transition metal materials, the copper-based sulfur compound Cu_*x*_S is widely used in tumor PTT due to its low toxicity, low cost, easy preparation, high photothermal conversion efficiency, and high thermal stability.^121^ CuS is also capable of mediating the Fenton reaction and has been developed for use in tumor CDT.^122,123^ To enhance the efficacy of PTT and immunotherapy for triple-negative breast cancer (TNBC), Cheng *et al.* developed a novel nanocomposite (AM@DLMSN@CuS/R848) based on the photothermal conversion properties of CuS nanomaterials.^124^ The nanocomposites were prepared by loading CuS and the immune adjuvant R484 into the pores of dendritic macroporous mesoporous silica (DLMSNs), wrapping the DLMSNs with breast cancer cell membranes, modifying the cell membrane surface with a polyethylene glycol junction with an acid-responsive benzoic-imine bond, and finally connecting the anti-PD-1 peptide4 AUNP-12 through the benzoic-imine bond. Under NIR irradiation, the wrapping of 4T1 cancer cell membranes endowed the nanocomposites with good TNBC targeting and could exert the efficacy of photothermal ablation. In the tumor microenvironment (TME), tumor antigens respond to the photothermal effect to produce and gradually release R848. The release of immune adjuvant could promote T-cell activation and significantly enhance the immune response of the body, while the acid-responsive benzoic–imine bond would rupture in the TME to release AUNP-12 for an immune checkpoint blocking assay (ICB). ICB inhibits T-cell apoptosis, blocks tumor cell immune escape, and prevents TNBC metastasis and recurrence.^125–128^ Thus, this novel nanomaterial significantly improved the immune efficacy of TNBC and ameliorated the problem of TME immunosuppression in addition to achieving PTT, which provides a promising strategy for tumor therapy.

**Fig. 8:**
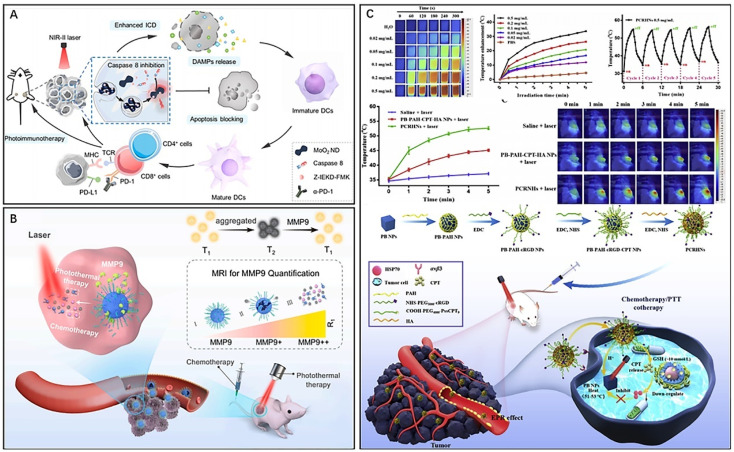
(A) MoO_2_ NDs for NIR-II photoimmunotherapeutic nanosystems. Reproduced from ref. 130 with permission from ACS, copyright 2022. (B) PMP@USPIO/DOX for synergetic chemo-photothermal therapy. Reproduced from ref. 140 with permission from Elsevier, copyright 2022. (C) A multifunctional nanoparticle system (PCRHN) for breast cancer therapy. Reproduced from ref. 142 with permission from Elsevier, copyright 2022.

In addition to Cu_*x*_S nanocomposites and carbon-based nanomaterials, two-dimensional transition metal oxides, such as MoO_2_, are also a promising class of PTAs for PTT of tumors due to their good biocompatibility and excellent photothermal conversion properties.^129^ Lan *et al.* utilized the photothermal properties of MoO_2_ for NIR II PTT (Fig. 8A).^130^ Compared with the NIR I region, NIR II PTT has stronger tissue penetration ability, enabling better photothermal ablation of deep tumors.^131^ Interestingly, researchers found that MoO_2_-mediated PTT also induced an immune response in the body, increasing the immune stimulation of tumor tissue and releasing a large number of damage-associated molecular patterns (DAMPs) through the process of immunogenic cell death (ICD) in the tumor tissue.^132^ These DAMPs promote the immune response through mutual recognition with macrophages, increase the infiltration of immune cells in the TME by promoting T-cell activation, and prevent the metastasis and spread of cancer by competitively binding to receptors on immune or tumor cells and blocking immune checkpoints (*e.g.*, aPD-L1) to avoid T-cell apoptosis and immune escape of cancer cells.^133^

In addition to MoO_2_, MoS_2_ is also a promising PTA for tumor PTT and PAI due to its excellent photothermal conversion properties.^134^ Hu *et al.* utilized MoS2's photothermal properties and multimodal imaging capabilities to design a tumor nanotherapeutic platform, 1-MT-Pt-PPDA@MoS_2_, which integrated three therapeutic modalities: PTT, immunotherapy, and chemotherapy. To enhance photothermal ablation and tumor thermography and PA imaging, the PDA coating was modified onto the MoS_2_ surface.^135^ Then, it was modified with methoxy-PEG-amine (mPEG-NH_2_) to increase the stability of the complex. Finally, the drugs 1-MT and cisplatin were loaded into MoS_2_ for PTT, immunotherapy, and chemotherapy combined therapy. 1-MT inhibits IDO-mediated tumor immunosuppressive responses and enhances the antitumour capacity of T cells. Moreover, PTT and chemotherapy induce ICD responses that promote T-cell infiltration in the TME and the body's immune response while inducing immune escape of tumor cells, leading to tumor metastasis and spread. However, 1-MT can function as an immune checkpoint blocker and inhibit T-cell apoptosis and tumor spread through ICB. Therefore, this novel nanocomposite platform could realize the visualization of tumors and the combination of PTT, immunotherapy and chemotherapy.

In addition, metallic materials such as Ag also have good photothermal conversion ability and can be well applied to PTT of tumors.^136^ For instance, Han *et al.* synthesized a multifunctional nanodrug delivery system (Ag_2_S-PAsp-cRGD) for PTT combined with immune targeting to address the complex tumor microenvironment.^137^ This nanosystem consists of Ag_2_S nanoparticles that were first modified with aspartic acid (PAsp) to construct a drug delivery platform for loading the chemotherapeutic drug DOX. Subsequently, active targeting ligands (cRGD) were coupled to the nanoparticles to enhance their drug delivery capability. The Ag_2_S nanoparticles produced heat to kill tumor cells, while the released DOX exerted chemotherapeutic effects. In addition, the acidic environment and high concentration of GSH in the tumor microenvironment were favorable for DOX release. This combination of PTT and chemotherapy could also induce immunogenic cell death (ICD), promote T-cell activation, and enhance the immune response of the body. The *in vitro* and *in vivo* experiments confirmed that this nanosystem has excellent photothermal antitumour effects.

Inorganic magnetic nanoparticles, such as superparamagnetic iron oxide (SPIO), have been widely used in cancer therapy due to their photothermal conversion properties and drug loading capacity.^138,139^ Additionally, SPIO has demonstrated excellent T2 contrast capabilities, while ultrasmall superparamagnetic iron oxide (USPIO) has been shown to significantly enhance T1 contrast during clustering. To advance this approach, Chen *et al.* developed a matrix metalloproteinase-9 (MMP9)-responsive T2-T1 switching nanoplatform (PMP@USPIO/DOX, PMPSD) for MRI imaging-guided PTT/chemotherapy combined therapy (Fig. 8B).^140^ The platform employed the photothermal properties of USPIO and its enhanced MRI contrast capabilities by coloading with the chemotherapeutic drug DOX in amphiphilic polymeric micelles (PMs) containing MMP9-sensitive peptides to responsive release USPIO and DOX. The changes in T1 and T2 signals directly respond to the content of MMP9, allowing for quantification and visualization of MMP9 in the TME. The platform provides a promising means for monitoring changes in the TME and drug release, facilitating disease assessment and diagnosis.

In addition to metallic inorganic photothermal conversion materials, Prussian blue (PB) is of great interest to researchers due to its excellent NIR photothermal conversion properties and drug loading capacity. PB has a simple synthesis process, low cost of raw materials, and high biocompatibility, making it a desirable PTA for tumor PTT.^141^ It is also one of the most widely used inorganic nanomaterials at present. Yang *et al.* developed a novel nanocomposite (PCRHN) for targeted tumor PTT and chemotherapy combined therapy by loading reduction-responsive chemotherapeutic drug camptothecin (CPT) precursors onto PB NPs and then modifying the surface of the above hybrids with a tumor-targeting peptide ring (Asp-d-Phe-Lys-Arg-Gly) (cRGD) and hyaluronic acid (HA) (Fig. 8C).^142^ CPT prodrugs with reduction-responsive ability could be released under TME-specific physiological conditions, while CPT loading can also inhibit the expression of heat shock protein 70 (HSP70), thereby improving the efficacy of photothermal ablation.^143^ The modification of targeting peptide cRGD and HA significantly enhanced the tumor-targeting ability and biocompatibility of PCRHNs, reducing the toxic side effects of the drug. The high affinity of cRGD for α_v_β_3_ integrins overexpressed in the TME allows for targeted delivery in the TME, and HA increases the biocompatibility of nanomaterials.^144^ In conclusion, PCRHNs offer high tumor targeting ability while achieving the dual therapeutic effect of PTT combined with chemotherapy.

## 4. Conclusions

As a selective and noninvasive therapeutic modality, PTT plays a crucial role in the diagnosis, treatment, and prevention of tumors, significantly advancing the application of nanomaterials in clinical disease management. In this review, we briefly introduce the fundamental principles of PTT, corresponding properties, and therapeutic efficacy of different photothermal nanomaterial types. The organic and inorganic nanomaterials have been extensively studied for their use in PTT.^145–147^ In terms of organic nanomaterials, porphyrin-based and polymer-based materials have shown promise due to their high photothermal conversion efficiency and ease of functionalization, respectively. ICG is another organic material that has been widely studied and has demonstrated good biocompatibility and specificity for cancer cells. PDA is a multifunctional organic nanomaterial that possesses not only excellent photothermal conversion ability but also good antibacterial and free radical scavenging abilities. Inorganic nanomaterials such as gold nanomaterials, copper sulfide nanoparticles, and carbon-based materials such as graphene and carbon nanotubes have also shown potential for using in photothermal therapy due to their high photothermal conversion efficiency and ease to functionalization. Gold nanomaterials, in particular, have been extensively studied and have demonstrated good biocompatibility and low toxicity. However, their high cost and potential accumulation in the liver and spleen still need to be addressed. Therefore, there are still some urgent problems to be solved in the clinical application of photothermal nanomaterials in PTT.

First, the most significant challenge of PTT is the limitation of the tissue penetration depth of NIR light, which is limited and often cannot reach deeper tissues. Consequently, PTAs cannot exert the normal photothermal effect, and tumor cells cannot be completely ablated. Researchers have attempted to develop PTAs with strong absorption in the NIR II region, which has deeper tissue penetration ability than NIR I PTAs, further achieving more efficient tumor ablation.^148^ Other researchers have tried irradiating tumor tissue with a fibre-optic NIR laser^149^ to improve the limitation of NIR light penetration depth.

Second, the choice of NIR light also plays an important role in the efficacy of PTT. Numerous studies have shown that there is a close correlation between laser output frequency, wavelength, irradiation time and photothermal ablation efficiency. Moreover, the nanomaterials with different surface properties, chemical compositions and functional groups, make them absorb different wavelengths.^6^ Therefore, selecting the appropriate wavelength is a prerequisite for PTT.

Third, the high temperature generated by the photothermal effect of PTA in tumor tissues may harm to neighboring normal tissue cells while killing tumor cells, and the initial high temperature may also induce a large amount of heat shock protein (HSP) expression in the body, increasing the heat resistance of tumor cells and reducing the efficacy of photothermal therapy.^150,151^ To overcome these problems, some researchers have worked to design mild PTT,^152,153^ which is expected to kill tumors at a lower temperature. Novel nanocomposites have also been designed that can inhibit the expression of HSPs and reduce the tolerance of tumor cells to high temperatures, further improving the efficacy of PTT.

Fourth, due to their low tumor targeting ability^154^ or biological barriers,^155,156^ some nanomaterials can accumulate in tumor tissues. To address these issues, researchers have devised several improved strategies to enhance the tumor targeting of nanomaterials. For instance, some researchers have wrapped a layer of temperature-sensitive responsive material on the surface of PTA, which allows the drug to be released selectively in tumor tissues. Other researchers have used the inherent properties of the tumor microenvironment (TEM) to design highly targeted PTAs. Modifying tumor-targeting molecules on the surface of PTA based on the receptor–ligand principle, such as folic acid molecules, has also been employed to achieve active tumor targeting. In addition, some studies have focused on surface charge regulation^157^ and NO gas therapy^158^ to increase the EPR effect to enhance the antitumour effect of nanosystems. To overcome biological barriers, researchers have attempted to use biological cell membrane-wrapped nanosystems, coating a cytomembrane on the surface of PTA, to make it more biocompatible and easier to penetrate the biological barriers for accumulation in the tumor tissue.

Fifth, the potential biotoxicity of nanomaterials and immunological rejection are significant concerns. Therefore, the cytotoxicity of gold nanoparticles has been extensively studied,^159,160^ with results indicating that their toxicity is size- and morphology dependent. Therefore, researchers have attempted to reduce their cytotoxicity by altering their size and morphology, but doing so also affects their photothermal conversion efficiency. Thus, precise synthesis and modification of gold nanoparticles are required to reduce cytotoxicity while maintaining photothermal conversion efficiency. Additionally, CNTs have been shown to have pulmonary toxicity,^161^ necessitating an accurate assessment of their *in vivo* biotoxicity before using them as photothermal agents.^162^ To enhance biocompatibility and reduce cytotoxicity, researchers have attempted to wrap a PEG coating around the surface of these nanomaterials. This coating reduces cytotoxicity while maintaining photothermal conversion efficiency. Although several strategies can reduce biotoxicity, the improvement of detection methods is required to monitor the metabolic and excretion processes of these materials *in vivo*.

Sixth, the physicochemical properties of nanomaterials can also influence their effectiveness in PTT applications.^163^ The surface properties of these materials, for example, can affect their biocompatibility. If they are unstable, they may agglomerate during blood circulation, causing embolism and endangering human life.^21^ Wrapping nanocoatings (*e.g.*, silica) around the surface of nanomaterials can improve their stability and avoid aggregation. Furthermore, alterations in the size of these materials can impact their photothermal effect and transport in the vasculature. The large sizes may reduce or eliminate their photothermal effect and pose a risk of blocking blood vessels. Therefore, when increasing the size of nanomaterials, their safety during blood transport and potential impact on metabolic processes in the liver and kidneys must be considered. Ideally, nanomaterials should have a diameter between 20 nm and 100 nm (ref. 21) to ensure effective accumulation in tumor tissues. To improve the performance of nanomaterials in PTT applications, new synthetic strategies should be designed and developed.

Despite achieving impressive therapeutic effects in tumor treatment, we do not yet fully understand the mechanism of action of PTA within the body and various biological systems. Safety risks, including blood circulation time, biotoxicity, immune elimination reactions, and the ability to be cleared by the body, require further investigation.

## List of abbreviation

PTT: Photothermal therapy
LEDs: Light-emitting diodes
RT: Radiation therapy
CDT: Chemodynamic therapy
PDT: Photodynamic therapy
NIR: Near-infrared light
PTA: Photothermal nanoagent
EPR: Enhanced penetration and retention effect
FL: Fluorescence imaging
PAI: Photoacoustic imaging
ICG: Indocyanine green
PDT: Photodynamic therapy
ICD: Immunogenic cell death
HSA: Human serum albumin
NRP-1: Neuropilin-1
GBM: Glioblastoma
GP: Glucose polymer
ABC: ATP-binding cassette
TAAs: Tumor-associated antigens
PAMPs: Pathogen-associated molecular patterns
ACQ: Aggregation-caused quenching
TPyP: Tetrachloroaurate and meso-tetra(pyridyl)porphyrin
CORMs: Carbon monoxide-releasing molecules
GSH: Glutathione
ROS: Reactive oxygen species
DAMPs: Damage-associated molecular patterns
ATP: Adenosine triphosphate
CRT: Calreticulin
HMGB1: High mobility histone B1
RbB: Rhodamine b
Pp18-lipids: Purpurin 18 conjugates and pure lipids
MRI: Magnetic resonance imaging
PPy: Polypyrrole
PDA: Polydopamine
PANI: Polyaniline
PNA: Poly (acrylic acid-*b-N*-isopropylamide-*b*-acrylic acid)
APS: Ammonium persulfate
CQ: Chloroquine
GO_*x*_: Glucose oxidase
HSPs: Heat shock proteins
PDDA: Poly (diallyldimethylammonium chloride)
HA: Hyaluronic acid
PMs: Polymeric micelles
PEG: Polyethylene glycol
TK: Thioketal
GA: Garcinia cambogia
Lipo: Liposomes
gel: Hydrogel
COF: Covalent organic frameworks
PAMAM-ICG: Poly(amidoamine) dendrimer-conjugated ICG
PEG-*b*-PCL: Poly (ethylene glycol)-*b*-poly(ε-caprolactone)
Ce6: Chlorin e6 (Ce6)
Pt: Platinum
LSPR: Local surface plasmon resonance
PTX: Paclitaxel
PCM: Phase change material
HIF-1a: Hypoxia-inducible factor-1a
MDR1: Multidrug resistance gene 1
PEG: Polyethylene glycol
HSPs: Heat shock proteins
TF: Tumour antigen
GBNMs: Graphene-based nanomaterials
CNTs: Carbon nanotubes
POD: Peroxidase
·OH: Hydroxyl radicals
SL: Sucrose laurate
PL: Peptide lipids
DTX: Docetaxel
Hep: Heparin
IDO: Indoleamine 2,3-dioxygenase
GO: Graphene oxide
DOX: Doxorubicin
SERS: Surface-enhanced Raman spectroscopy
4-MPBA: 4-Mercaptophenylboronic acid
TNBC: Triple-negative breast cancer
TME: Tumor microenvironment
ICB: Immune checkpoint blocking assay
DAMPs: Damage-associated molecular patterns
ICD: Immunogenic cell death
aPD-L1: Anti-PD-L1
SPIO: Superparamagnetic iron oxide
USPIO: Ultrasmall superparamagnetic iron oxide
MMP9: Matrix metalloproteinase-9
PMs: Polymeric micelles
PB: Prussian blue
CPT: Camptothecin
HA: Hyaluronic acid
HSP70: Heat shock protein 70

## Author contributions

Shufan Duan and Yanling Hu: conceptualization, writing – original draft. Zhijing Zhang and Zilu Liu: investigation, formal analysis, visualization, copyright application. Ying Wang and Haiyang Guo: formal analysis, visualization. Ying Zhao and Kaiyuan Tang: writing – review & editing. Shengke Li: writing – review & editing. Yuchen Miao and Hengda Du: investigation, visualization. Dongliang Yang and Junjie Zhang: conceptualization, formal analysis, project administration, resources, writing – original draft, writing – review & editing, funding acquisition.

## Conflicts of interest

The authors declare that the research was conducted in the absence of any commercial or financial relationships that could be construed as a potential conflict of interest.

## Supplementary Material
